# Time to recovery and its predictors among adults hospitalized with COVID-19: A prospective cohort study in Ethiopia

**DOI:** 10.1371/journal.pone.0244269

**Published:** 2020-12-30

**Authors:** Saro Abdella Abrahim, Masresha Tessema, Atkure Defar, Alemayehu Hussen, Eshetu Ejeta, Getachew Demoz, Addisu Birhanu Tereda, Enatenesh Dillnessa, Altaye Feleke, Misiker Amare, Frehiwot Nigatu, Yaregal Fufa, Hailu Refera, Ayalew Aklilu, Munir Kassa, Tsigereda Kifle, Susan Whiting, Getachew Tollera, Ebba Abate

**Affiliations:** 1 Ethiopian Public Health Institute, Addis Ababa, Ethiopia; 2 Eka Kotebe General Hospital, COVID-19 Isolation and Treatment Center, Addis Ababa, Ethiopia; 3 International Institute for Primary health care- Ethiopia, Addis Ababa, Ethiopia; 4 ICAP-Ethiopia, Addis Ababa, Ethiopia; 5 Federal Ministry of Health, Addis Ababa, Ethiopia; 6 College of Pharmacy and Nutrition, University of Saskatchewan, Saskatoon, Canada; Azienda Ospedaliero Universitaria Careggi, ITALY

## Abstract

**Background:**

Various factors may determine the duration of viral shedding (the time from infection to viral RNA-negative conversion or recovery) in COVID-19 patients. Understanding the average duration of recovery and its predictors is crucial in formulating preventive measures and optimizing treatment options. Therefore, evidence showing the duration of recovery from COVID-19 in different contexts and settings is necessary for tailoring appropriate treatment and prevention measures. This study aimed to investigate the average duration and the predictors of recovery from Severe Acute Respiratory Syndrome Coronavirus 2 (SARS-CoV-2) infection among COVID-19 patients.

**Method:**

A hospital-based prospective cohort study was conducted at Eka Kotebe General Hospital, COVID-19 Isolation and Treatment Center from March 18 to June 27, 2020. The Center was the first hospital designated to manage COVID-19 cases in Ethiopia. The study participants were all COVID-19 adult patients who were admitted to the center during the study period. Follow up was done for the participants from the first date of diagnosis to the date of recovery (negative Real-time Reverse Transcriptase Polymerase Chain Reaction (rRT-PCT) test of throat swab).

**Result:**

A total of 306 COVID-19 cases were followed up to observe the duration of viral clearance by rRT-PCR. Participants’ mean age was 34 years (18–84 years) and 69% were male. The median duration of viral clearance from each participant’s body was 19 days, but the range was wide: 2 to 71 days. Cough followed by headache was the leading sign of illness among the 67 symptomatic COVID-19 patients; and nearly half of those with comorbidities were known cancer and HIV/AIDS patients on clinical follow up. The median duration of recovery from COVID-19 was different for those with and without previous medical conditions or comorbidities. The rate of recovery from SARS-CoV-2 infection was 36% higher in males than in females (p = 0.043, CI: 1.01, 1.85). The rate of recovery was 93% higher in those with at least one comorbidity than in those without any comorbidity. The risk of delayed recovery was not influenced by blood type, BMI and presence of signs or symptoms. The findings showed that study participants without comorbidities recovered more quickly than those with at least one comorbidity. Therefore, isolation and treatment centers should be prepared to manage the delayed stay of patients having comorbidity.

## Introduction

Understanding the factors associated with the duration of viral ribonucleic acid (RNA) shedding, the time from infection to viral RNA-negative conversion in patients with Coronavirus disease 2019 (COVID-19), is crucial in formulating preventive measures and optimizing treatment options [1]. Centers for Disease Control and Prevention (CDC) recommends two consecutive negative results of SARS-CoV-2 by Real-time Reverse Transcriptase Polymerase Chain Reaction (rRT-PCR) in 24 hours to conclude a patient’s recovery from COVID-19 [2].

The median duration of viral shedding in COVID-19 patients ranges from 8–30 days while the longest duration was reported as 47 days [1, 3–8]. Factors that are associated with prolonged duration of viral RNA shedding in COVID-19 patients include older age, a time lag from illness onset to hospital admission, diarrhea, corticosteroid treatment and lopinavir/ritonavir use [1]. In contrast, a study reported that antiviral therapy and corticosteroid treatment were not independent factors for prolonged duration of viral shedding [7]. Further, a study in Japan showed that fever and time from illness onset to hospitalization were associated with increased odds of prolonged duration of viral shedding [9]. Male sex, immunoglobulin use, Acute Physiology and Chronic Health Evaluation II (APACHE II) score, and lymphocyte count were independent factors associated with a prolonged duration of SARS-CoV-2 shedding in a study conducted among hospitalized patients with COVID-19 in Zhejiang Province, China [8]. High Body Mass Index (BMI) was also reported to be associated with a longer duration of viral shedding in COVID -19 patients in a study conducted in Italy [10]. A study by Xu revealed that severe illness requiring invasive mechanical ventilation was an independent factor for prolonged SARS-CoV-2 RNA shedding [11], while another study reported no correlation between severity and viral shedding [4].

Various factors determine the duration of the viral shedding in COVID-19 patients. Therefore, evidences that show the duration of recovery from COVID-19 in different contexts and settings are necessary to tailor appropriate treatment and prevention measures.

Most of the published studies conducted on the duration of SARS-COV-2 shedding among COVID-19 patients are from China and Europe. There are few studies conducted in Africa where the epidemic seems to be different from that of other continents with regards to speed of the spread of the virus and the death toll recorded. Hence, this study aimed to investigate predictors of recovery from SARS-CoV-2 infection among COVID-19 patients admitted to Eka Kotebe General Hospital, Isolation and Treatment Center, Addis Ababa, Ethiopia.

## Method

### Study design and settings

A hospital-based prospective cohort study involving 306 COVID-19 cases was conducted at Eka Kotebe General Hospital, COVID-19 Isolation and Treatment Center. The Center was the first hospital designated to manage positive COVID-19 cases in Ethiopia. It had a capacity of admitting 600 cases. The Center is located in the capital city of Ethiopia, Addis Ababa, where the largest airport in country is located. The isolation and treatment center was the place where all Ethiopian and none-Ethiopian citizens with COVID-19 were admitted for isolation, care and support.

### Study participants

The study participants were all COVID-19 adult patients who were admitted to the Eka Kotebe General Hospital, COVID-19 Isolation and Treatment Center from March to June 2020. Regardless of sign or symptom development, all individuals with confirmed SARS-CoV-2 infection were obliged to be admitted to the center during the study period.

### Sampling and study period

All COVID-19 cases who were admitted to the Eka Kotebe General Hospital, COVID-19 Isolation and Treatment Center during the study period and who gave consent to participate in the study were included. The study enrolled cases between the month of March and May 2020 and followed up to their respective date of rRT-PCR negative result for SARS-CoV-2.

The sample size for duration of clearance was calculated taking the following assumptions in to consideration:

- α = 0.05,: 95% Confidence Interval (CI)
- β = 0.2, 90% power
- 50% of subjects in the control group survive by the end of the study (no literature found to reveal this variable before the study time)
- equal proportion of individuals in each group
- 10% withdrawal

The minimum sample size required to conduct this study was 246. This study has enrolled and followed 306 participants. This increases generalizability of the findings to COVID-19 patients in the country.

### Operational definitions

COVID-19 cases are all individuals tested infected with SARS-CoV-2 by rRT-PCR.

A symptomatic case is defined as any SARS-CoV-2 positive individual by rRT-PCR with at least one sign or symptom for COVID-19 including but not limited to: cough, fever, headache, muscle pain and shortness of breath

Cases with comorbidity are those COVID-19 patients with at least one known preexisting medical illness

Duration of recovery is defined as the number of days between the first rRT-PCR positive test for SARS-CoV-2 and two consecutive negative results of the virus by the rRT-PCR in 24 hours

### Variables

Explanatory variables were age, sex, BMI, blood group, symptoms and comorbidities. BMI was calculated as weight in kilograms divided by height in meters squared. Both height and weight of patients were taken on admission to the hospital.

The outcome variable was duration/time of recovery from COVID-19. The National Comprehensive COVID-19 Management Guide document developed by The Ethiopian Ministry of Health in April 2020 strongly recommend admission or isolation of all laboratory confirmed SARS-CoV-2 infected individuals as soon as possible. All study participants were admitted to the hospital in 24 hours after testing positive for SARS-CoV-2.

### Testing schedule and procedure

After the fifth day of admission and in 72 hours intervals thereafter, throat swabs were collected for each participant to test for SARS-CoV-2 using rRT-PCR. If a test result for the SARS-CoV-2 turned negative, the test would be repeated after 24 hours to confirm recovery. Those with two consecutive negative rRT-PCR test results in 24 hours were considered free from the virus and discharged from the hospital.

For those study participants whose SARS-CoV-2 test result persisted to be positive after the fifth day of admission, testing for the virus would be continued every 72 hours until two consecutive negative results in 24 hours were obtained.

### Data management

A log sheet to record patients’ SARS-CoV-2 test results by rRT-PCTR was prepared. Physicians at the Eka Kotebe General Hospital, COVID-19 Isolation and Treatment Center recorded the COVID-19 laboratory results of all patients. Onsite data entry was conducted using tablets by trained data collectors in the facility, and data were transferred to the Ethiopian Public Health Institute Server via the REDCap system.

Continuous variables were expressed as mean ± standard deviation (SD) for the normally distributed data or median with interquartile (IQR) for skewed data. Descriptive analysis of survival data was presented graphically using Kaplan-Meier (KM) estimator. Log-Rank test was used to compare the survival experience of different category of covariates. The proportional hazard assumption (PHA) was checked using the Schoenfeld residual test. Cox proportional-hazards regression model was used to identify the potential risk factors associated with the duration time of recovery among COVID-19 cases. Statistical significance was defined as P<0.05. All analyses were done with STATA version 16.1 software.

### Ethical clearance

The study protocol was developed by some members of study team and reviewed by the Ethiopian Public Health Institute’s Institutional Review Board. The protocol was approved by the IRB (Ethics Ref. No. EPHI 6.13/690). Written informed consent of every participating case was obtained. Data security and participants’ confidentiality were maintained at all levels of data management. All study participants were adult (≥ 18 years).

## Result

A total of 306 COVID-19 cases were followed up to observe duration of viral clearance by rRT-PCR. The participants’ mean age was 34 years (range, 18–84 years) and 69% were male. Around 26% of the participants had BMI ≥ 25, with a range of 25.0 to 50.39. The majority of the participants (41%) had blood type O and only 10% had blood type AB. Thirteen percent and 22% of the cases had comorbidities and COVID-19 related signs or symptoms, respectively (Table 1).

**Table 1:** Background characteristics of study participants admitted at the Eka Kotebe General Hospital, COVID-19 Isolation and Treatment Center, 2020.

Variables	Category	Frequency (n)	Percent (%)
**Age (y)**	All, min = 18, max = 84	33.74±13.34	
**Sex**	Female	95	31.05
	Male	211	68.95
**BMI**	<25, min = 16.22	219	73.49
	≥25, max = 50.39	79	26.51
**Blood type**	A	61	27.23
	B	49	21.88
	AB	23	10.27
	O	91	40.63
**Comorbidities**	Have at least one comorbidity	41	13.40
	Have no comorbidity	265	86.60
**Symptom status**	Have ≥ 1 symptom	67	22.26
	Have no symptom	234	77.74

**Fig 1:**
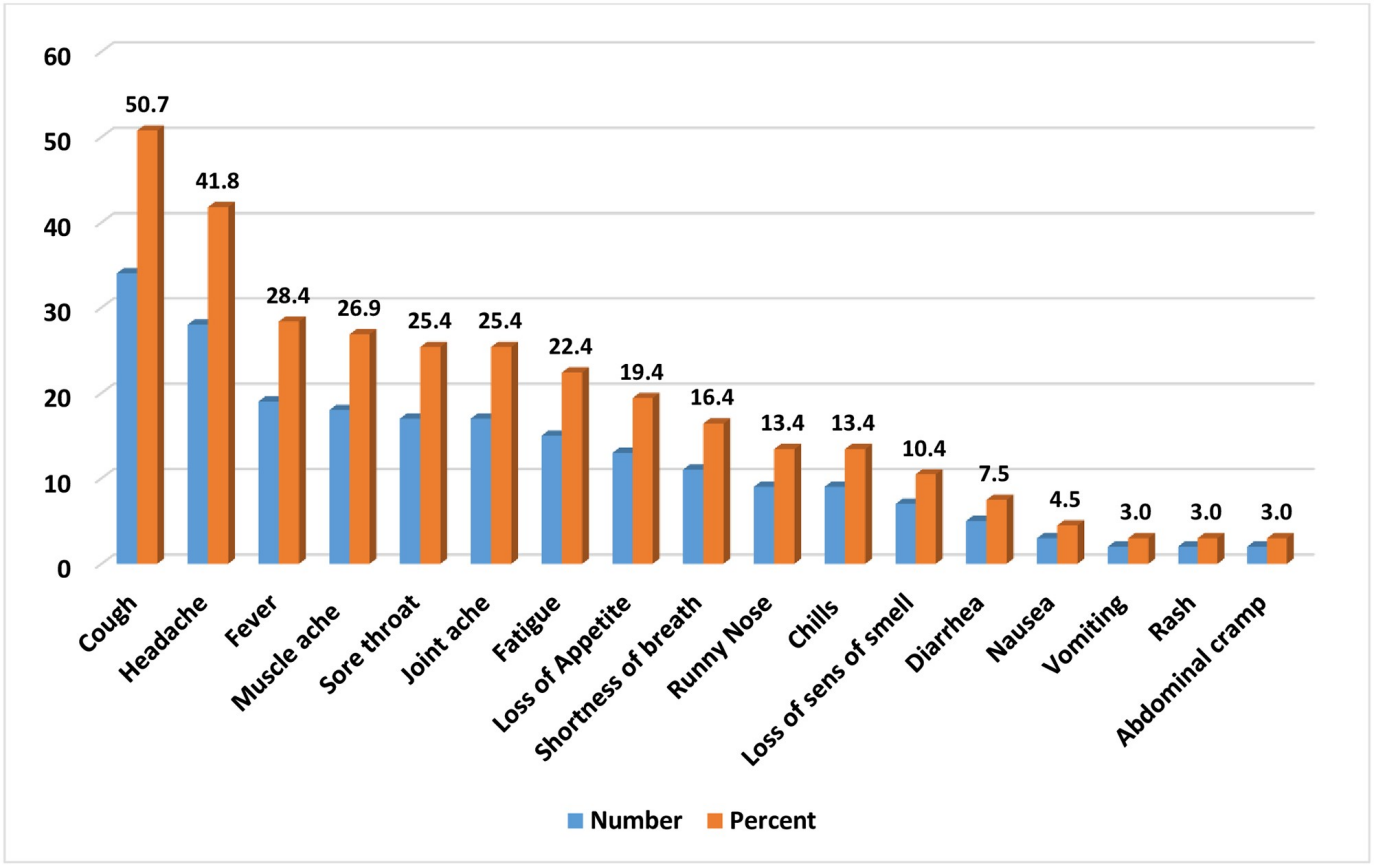
Signs and symptoms observed in the study participants at Eka Kotebe General Hospital, COVID-19 Isolation and Treatment Center, 2020.

Cough followed by headache was the leading sign of COVID-19 in the 67 symptomatic COVID-19 patients in the study. Vomiting, rash and abdominal cramping were the least observed signs and symptoms (Fig 1).

**Fig 2:**
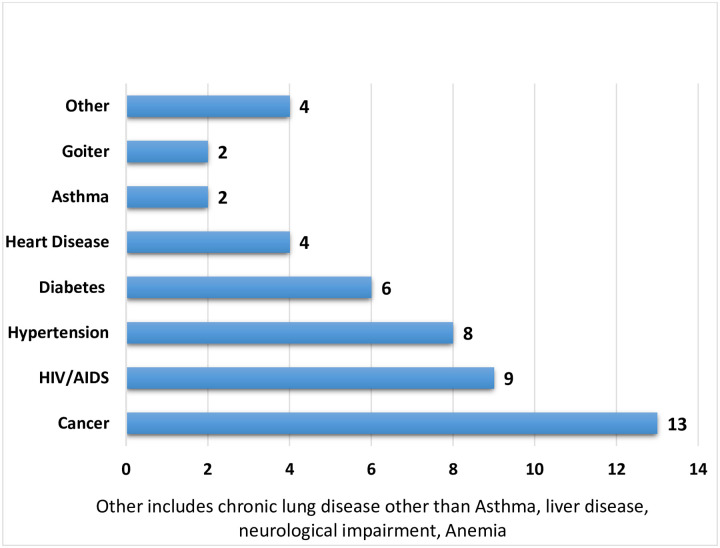
Number of study participants with preexisting medical conditions, Eka Kotebe General Hospital, COVID-19 Isolation and Treatment Center, 2020.

Nearly half of the study participants with comorbidities were known cancer and HIV/AIDS patients on treatment and care follow up (self-reported). The second and third predominant comorbidities were hypertension and diabetes mellitus (Fig 2).

The median duration of viral clearance from each participant’s body was 19 days, but the range was wide (2 to 71 days). The median duration of a negative result for SARS-CoV-2 by PCR for participants with different sex, BMI, blood type and symptom status was similar. Nonetheless, the date of recovery from the virus was different for those with and without previous medical conditions or comorbidities (Table 2).

**Table 2:** Duration of viral clearance by different factors: Eka Kotebe General Hospital, COVID-19 Isolation and Treatment Center, 2020.

Variable	Category	Median duration of negative result PCR (days)	Log-rank test, P-value	95% CI
**All**	Min = 2, Max = 71	19		
**Sex**	Female	21	0.2673	18, 23
	Male	18		15, 20
**BMI**	<25	20	0.8253	18, 21
	>25	18		15, 21
**Blood type**	A	20	0.5975	15, 22
	B	20		16, 23
	AB	14		14, 19
	O	19		15, 21
**Comorbidities**	Have at least one comorbidity	16	0.0020	14, 19
	Have no comorbidity	20		18, 21
**Sign or Symptom status**	Have symptom	20	0.8108	16, 22
	Have no symptom	19		16, 21

The highest average duration of viral clearance was observed in Diabetic patients (23 days) and the lowest average duration was observed in known Cancer and HIV patients (14 days).

**Fig 3:**
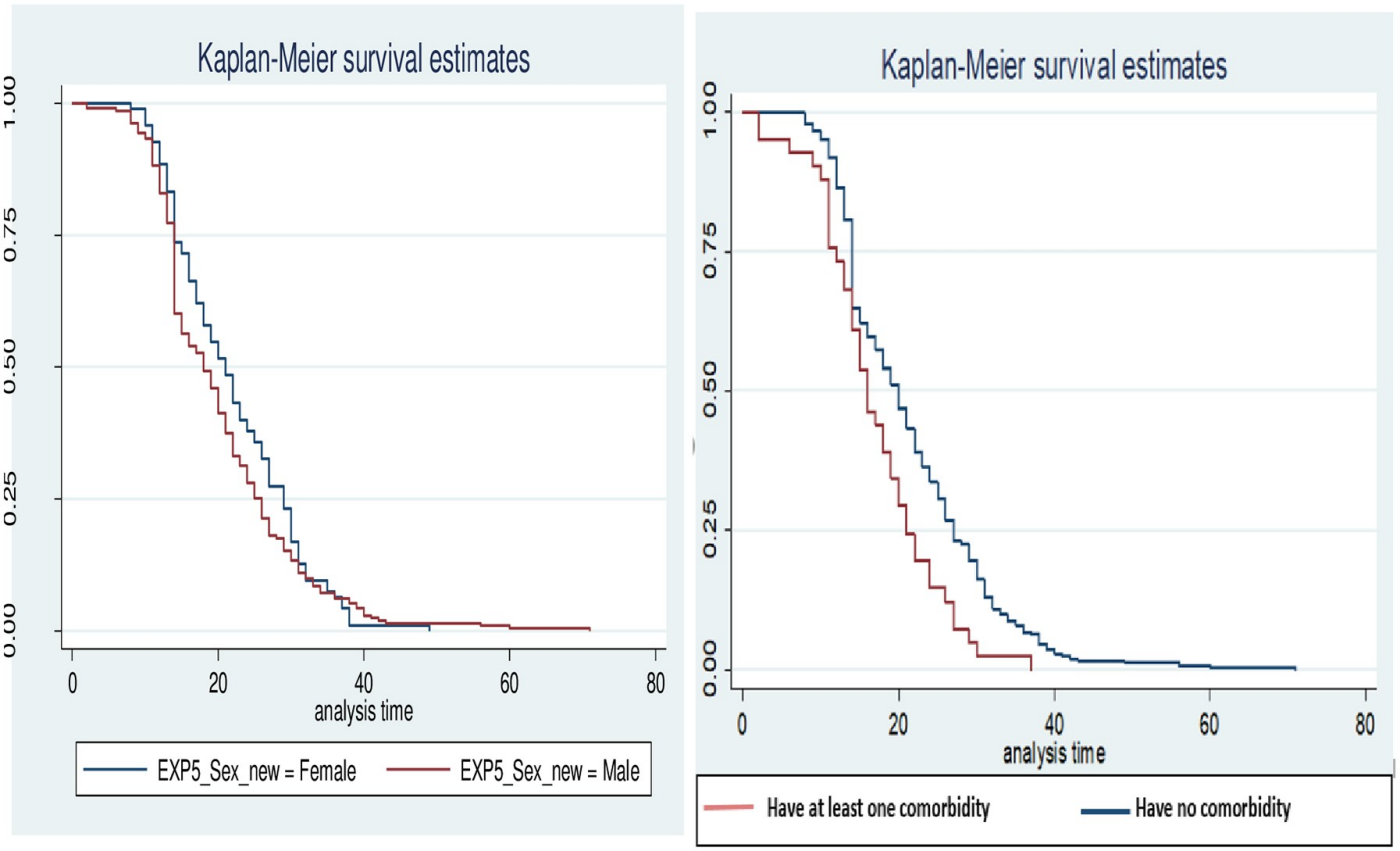
Kaplar-Meier survival estimate by comorbidity and sex.

Duration of recovery from COVID-19 was plotted using Kaplar-Meier survival estimates (Fig 3). It shows that there was no significant difference in the median duration of viral clearance between males and females. However, there was a significant difference in the median duration of viral clearance between those patients with and without at least one comorbidity.

The rate of recovery from SARS-CoV-2 infection was 36% higher in males than in females (p = 0.043, CI: 1.01, 1.85). The rate was 93% higher in those with at least one comorbidity than in those without any comorbidity. The risk of delayed recovery was not influenced by blood type, BMI and existence of signs and symptoms (Table 3).

**Table 3:** Cox proportional hazards analysis of predictors of delayed recovery of patients with corona virus disease 2019 (n = 223).

Variable	Category	AHR (95% CI)	p-value
**Age**	33.74±13.34	1.0 (0.99, 1.01)	0.992
**Sex**			
	Male	**1.36 (1.01, 1.85)**	0.045
	Female	1	
**Comorbidity**			
	No comorbidity	1	
	At least one comorbidity	**1.93 (1.23, 3.04)**	0.004
**Blood Group**	A	1	
	B	0.80 (0.54, 1.19)	0.277
	AB	1.06 (0.65, 1.73)	0.825
	O	0.84 (0.60, 1.16)	0.287
**BMI**	<25	1.22 (0.88, 1.69)	0.225
	> = 25	1	
**Patient symptoms**	Have no symptom	1.20 (0.84, 1.70)	0.314
	Have symptom	1	

## Discussion

This study demonstrated that the median duration of SARS-CoV-2 clearance from study participants’ body was 19 days. It also indicates that having one or more comorbidity significantly delayed the viral clearance from the body. However, BMI, age, sex and blood group were not significantly associated with the time of viral clearance.

The median duration of viral clearance (19 days) of this study was consistent with some of the studies done in China. The average duration of the viral clearance was reported to be 21 days with 95% (CI 20–22 days) in Shenzhen and 20 days (IQR 17–24) in Wuhan [12, 13]. Moreover, our finding was also similar to a study conducted to determine viral load in upper respiratory specimens of infected patients where the average duration of SARS-CoV-2 viral clearance was 21 days [14]. However, the median duration of viral clearance in many studies was lower. For instance, a study done in Israeli (13.2 days) [15], Singapore (12 days) [16], Wuhan (17 days) [17], Shanghai (11 days) [18], Jiangsu and Anhui (11 days) [19], Shandong (14 days) [20] and Guangzhou (12 days) [21]. The discrepancy between studies might be attributed to the differences in disease severity, sample size, study setting, socioeconomic conditions, and type of specimen for testing.

In this study, we found a significant association between sex of the study participants and delayed viral clearance/time to recovery. Males had 36% higher risk of delayed viral clearance as compared with females. This finding was in line with studies conducted in Shenzhen [12], Israeli [15], and Wuhan [11] that reported males were more likely to have delayed viral clearance duration. According to Xu et al., the reasons sex-related difference in SARS-CoV-2 infection is not clear [11]. Some studies have indicated that sex-related differences might be confounded by comorbidity and females being more immune-privileged than males that might be related to sex hormones with immune-enhancing effect like estrogen [11, 22, 23].

Our study further revealed that SARS-CoV-2 viral clearance was more likely to be delayed among COVID-19 patients with at least one comorbidity compared to those without comorbidity. Several studies also reported that comorbidity was an independent risk factor that can delay viral clearances [19, 20, 24] while other studies reported no significant association between comorbidity and viral clearance [5, 17, 21]. There may be differences due to the nature of treatment for comorbidities and/or whether patients had been compliant with treatment regimens or not.

In line with other studies [5, 17], this study found no association between age and viral clearance. Some studies reported older age was independently associated with delayed clearance of SARS-COV-2 [1, 17, 20]. This might be attributed to the degeneration of physiological functions and low immune status among elders. An older aged person with COVID-19 has poor clinical outcomes because T-cell numbers and functions are compromised with aging, resulting in less control of viral replication. Moreover, older patients are more likely to exhibit severe comorbidity than younger adults [13, 25, 26]. It should be noted that less than 5% of our study participants were 65 years old and above.

Obesity had been reported as a risk factor for severe COVID-19 infection [27]. However, in our study, no relationship was found between time to viral clearance and BMI. A study done in Italy found that obesity (BMI > 30) was related to longer hospital stay and longer time to negative swab for SARS-CoV-2 [10]. Others have reported that high BMI was significantly associated with positive test, and risk of death related to COVID-19 in the United Kingdom, and that this relationship was stronger in non-white groups [27]. A larger patient group may be needed to test for a body weight effect in Ethiopian patients.

In this study, no blood type was significantly associated with the viral clearance of SARS-CoV-2. This finding is consistent with a systematic review and meta-analysis study that reported no significant association between blood groups and recovery from COVID-19 [28]. Other studies also reported that blood type was not associated with the risk of progression of the disease to a severe infection defined as intubation and death [29–31, 32, 33]. Nevertheless, there are data that blood type plays a role in disease acquisition and severity of other diseases [30, 34].

Our study has some limitations related to the study design, particularly lead time bias. Participants in younger and older age groups were not adequately represented. Participants made self-report of preexisting medical conditions that were considered as comorbidity in the analyses.

### Conclusion and recommendations

The findings of this study show that the study participants without comorbidities recovered more quickly than other participants with at least one comorbidity. Therefore, COVID-19 patients with comorbidities will be isolated for a longer time than those patients without comorbidity. Thus, isolation and treatment centers should be prepared for the delayed stay of COVID-19 patients with comorbidities. Further, considering the delay in recovery, those in the population with comorbidities should be given priority in shielding them from contracting the SARS-CoV-2.

## Supporting information

S1 Data (XLSX)
